# Noninvasive ventilation vs. high-flow nasal cannula oxygen for preoxygenation before intubation in patients with obesity: a post hoc analysis of a randomized controlled trial

**DOI:** 10.1186/s13613-021-00892-8

**Published:** 2021-07-22

**Authors:** Maeva Rodriguez, Stéphanie Ragot, Rémi Coudroy, Jean-Pierre Quenot, Philippe Vignon, Jean-Marie Forel, Alexandre Demoule, Jean-Paul Mira, Jean-Damien Ricard, Saad Nseir, Gwenhael Colin, Bertrand Pons, Pierre-Eric Danin, Jérome Devaquet, Gwenael Prat, Hamid Merdji, Franck Petitpas, Emmanuel Vivier, Armand Mekontso-Dessap, Mai-Anh Nay, Pierre Asfar, Jean Dellamonica, Laurent Argaud, Stephan Ehrmann, Muriel Fartoukh, Christophe Girault, René Robert, Arnaud W. Thille, Jean-Pierre Frat, REVA Network, Delphine Chatellier, Florence Boissier, Anne Veinstein, René Robert, Claire Dahyot-Fizelier, Auguste Dargent, Audrey Large, Emmanuelle Begot, Claire Mancia, Maxence Decavele, Martin Dres, Samuel Lehingue, Laurent Papazian, Marine Paul, Nathalie Marin, Matthieu Le Meur, Mohammed Laissy, Anahita Rouzé, Matthieu Henry-Lagarrigue, Aihem Yehia, Frédéric Martino, Charles Cerf, Pierre Bailly, Julie Helms, Jean Baptiste Putegnat, Keyvan Razazi, Thierry Boulain, Pierre Asfar, Séverin Cabasson, Florent Wallet, Kada Klouche, Frédéric Bellec

**Affiliations:** 1 Médecine Intensive Réanimation, CHU de Poitiers, Poitiers, France; 2 INSERM, CIC-1402 ALIVE, University of Poitiers, Poitiers, France; 3 INSERM, CIC-1402, Biostatistics, Université de Poitiers, Faculté de Médecine Et de Pharmacie de Poitiers, Poitiers, France; 4 Service de Médecine Intensive Réanimation, CHU Dijon Bourgogne, Dijon, France; 5 Université Bourgogne Franche-Comté Lipness Team UMR 1231 Et INSERM CIC 1432 Epidémiologie Clinique, Dijon, France; 6 Réanimation Polyvalente, CHU Dupuytren, 87042 Limoges, France; 7 Clinical Investigation Centre INSERM 1435, 87042 Limoges, France; 8 Médecine Intensive Réanimation Détresses Respiratoires Et Infection Sévères, AP-HM, CHU Nord and CEReSS - Center for Studies and Research On Health Services and Quality of Life EA3279, Aix-Marseille University, Marseille, France; 9 AP-HP 6, Groupe Hospitalier Pitié-Salpêtrière Charles Foix, Service de Pneumologie Et Réanimation Médicale du Département R3S, Paris, France; 10 INSERM, UMRS1158 Neurophysiologie Respiratoire Expérimentale Et Clinique, Sorbonne Université, Paris, France; 11 Assistance Publique des Hôpitaux de Paris, Groupe Hospitalier Universitaire de Paris Centre, Hôpital Cochin, Réanimation médicale, Paris, France; 12 Faculté de Médecine, Université Paris Descartes, Paris, France; 13 AP-HP, Hôpital Louis Mourier, Service de Réanimation Médico-Chirurgicale, 92700 Colombes, France; 14 UMR IAME 1137, Université Paris Diderot, Sorbonne Paris Cité, 75018 Paris, France; 15 INSERM, IAME 1137, 75018 Paris, France; 16 Médecine Intensive-Réanimation, CHU de Lille, Inserm U1285, Univ. Lille, CNRS, UMR 8576 - UGSF - Unité de Glycobiologie Structurale Et Fonctionnelle, 59000 Lille, France; 17 Centre Hospitalier Départemental de La Roche Sur Yon, Service de Réanimation Polyvalente, La Roche sur Yon, France; 18 Service de Réanimation, CHU Point-À-Pitre, Pointe-à-Pitre, Guadeloupe France; 19 Réanimation Chirurgicale, CHU de Nice, Nice, France; 20 INSERM U1065, team 8, C3M, Nice, France; 21 Réanimation polyvalente, Hôpital Foch, Suresnes, France; 22 Service de Réanimation Médicale, CHU de La Cavale Blanche, Brest, France; 23 Faculté de Médecine, Hôpitaux universitaires de Strasbourg, Service de Médecine Intensive-Réanimation, Nouvel Hôpital Civil, Université de Strasbourg (UNISTRA), Strasbourg, France; 24 INSERM (French National Institute of Health and Medical Research), UMR 1260, Regenerative Nanomedicine (RNM), FMTS, Strasbourg, France; 25 Réanimation Chirurgicale, CHU de Poitiers, Poitiers, France; 26 Service de Réanimation Polyvalente, Centre Hospitalier Saint Joseph-Saint Luc, Lyon, France; 27 Assistance Publique des Hôpitaux de Paris, CHU Henri Mondor, DHU A-TVB, Service Médecine Intensive Réanimation Médicale, 94010 Créteil, France; 28 Faculté de Médecine de Créteil, Groupe de Recherche Clinique CARMAS, Université Paris Est Créteil, 94010 Créteil, France; 29 INSERM, Unité UMR 955, IMRB, 94010 Créteil, France; 30 Service de Médecine Intensive Réanimation, Centre Hospitalier Régional D’Orléans, Orléans, France; 31 Département de Médecine Intensive-Réanimation, CHU D’Angers, Angers, France; 32 Médecine Intensive Réanimation, CHU de Nice, Nice, France; 33 UR2CA, Université Cote D’Azur, Nice, France; 34 Service de Réanimation Médicale, Hospices Civils de Lyon, Groupement Hospitalier Universitaire Edouard Herriot, 69003 Lyon, France; 35 CHRU de Tours, Médecine Intensive Réanimation, CIC1415,, CRICS-TriggerSEP Research Network, Tours, France; 36 Centre D’Etudes Des Pathologies Respiratoires, INSERM U1100, Université de Tours, Tours, France; 37 Assistance Publique – Hôpitaux de Paris, Hôpital Tenon, Service de Médecine Intensive Réanimation, Sorbonne Université, 75020 Paris, France; 38 CHU de Rouen, Normandie Univ, UNIROUEN, Department of Medical Intensive Care, Charles Nicolle University, Hospital, Rouen, France; 39 EA3830-GRHV, Institute for Research and Innovation in Biomedicine (IRIB), Rouen University, 76000 Rouen, France

**Keywords:** Preoxygenation, Intubation, Non-invasive ventilation, High-flow oxygen, Respiratory failure, Obesity, Hypoxemia

## Abstract

**Background:**

Critically ill patients with obesity may have an increased risk of difficult intubation and subsequent severe hypoxemia. We hypothesized that pre-oxygenation with noninvasive ventilation before intubation as compared with high-flow nasal cannula oxygen may decrease the risk of severe hypoxemia in patients with obesity.

**Methods:**

Post hoc subgroup analysis of critically ill patients with obesity (body mass index ≥ 30 kg·m^−2^) from a multicenter randomized controlled trial comparing preoxygenation with noninvasive ventilation and high-flow nasal oxygen before intubation of patients with acute hypoxemic respiratory failure (PaO_2_/FiO_2_ < 300 mm Hg). The primary outcome was the occurrence of severe hypoxemia (pulse oximetry < 80%) during the intubation procedure.

**Results:**

Among the 313 patients included in the original trial, 91 (29%) had obesity with a mean body mass index of 35 ± 5 kg·m^−2^. Patients with obesity were more likely to experience an episode of severe hypoxemia during intubation procedure than patients without obesity: 34% (31/91) vs. 22% (49/222); difference, 12%; 95% CI 1 to 23%; *P* = 0.03. Among patients with obesity, 40 received preoxygenation with noninvasive ventilation and 51 with high-flow nasal oxygen. Severe hypoxemia occurred in 15 patients (37%) with noninvasive ventilation and 16 patients (31%) with high-flow nasal oxygen (difference, 6%; 95% CI − 13 to 25%; *P* = 0.54). The lowest pulse oximetry values during intubation procedure were 87% [interquartile range, 77–93] with noninvasive ventilation and 86% [78–92] with high-flow nasal oxygen (*P* = 0.98). After multivariable analysis, factors independently associated with severe hypoxemia in patients with obesity were intubation difficulty scale > 5 points and respiratory primary failure as reason for admission.

**Conclusions:**

Patients with obesity and acute hypoxemic respiratory failure had an increased risk of severe hypoxemia during intubation procedure as compared to patients without obesity. However, preoxygenation with noninvasive ventilation may not reduce this risk compared with high-flow nasal oxygen.

*Trial registration* Clinical trial number: NCT02668458 (http://www.clinicaltrials.gov)

**Supplementary Information:**

The online version contains supplementary material available at 10.1186/s13613-021-00892-8.

## Background

The prevalence of obesity has dramatically increased around the world and can affect up to 20% of patients in intensive care units (ICUs) [1–3]. Complications during intubation procedure are particularly frequent in this population and may lead to severe hypoxemia episodes [3–6]. Cardiac arrest is the ultimate complication of severe hypoxemia during intubation procedure occurring in 2–3% in ICU, and it is strongly related to hypoxemia, overweight and obesity [7, 8]. Lung function disorders, characterized by reduction in lung volumes and greater atelectasis formation, may explain the risk of severe hypoxemia in patients with obesity [4, 9–13].

Noninvasive ventilation and high-flow nasal cannula oxygen therapy are two oxygen supports largely used in ICUs to manage patients with hypoxemic respiratory failure or to prevent reintubation during the post-extubation period [14–19]. These two techniques have been proposed as an alternative to standard oxygen preoxygenation using valve-bag mask to optimize preoxygenation before intubation of hypoxemic patients in ICUs [20–28]. Noninvasive ventilation and high-flow nasal oxygen provide a positive end-expiratory pressure (PEEP) and a higher fraction of inspired oxygen (FiO_2_) leading to better blood oxygenation than does standard oxygen [29–32]. In fact, noninvasive ventilation helps in higher oxygenation than high-flow nasal cannula oxygen probably favored by a higher PEEP effect [31, 33]. However, during intubation procedure, high-flow nasal cannula oxygen may have an additional theoretical advantage, which is the maintenance of oxygenation during the apneic phase of intubation after anesthetic induction [34], thereby avoiding hypoxemia, whereas noninvasive ventilation is removed at this phase. Several studies in critically ill patients have shown that these two strategies of preoxygenation may improve oxygenation and prevent complications (episodes of hypoxemia or cardiac events) during intubation procedure as compared to standard oxygen preoxygenation [23, 25, 27, 28]. A recent multicenter randomized controlled trial did not show any difference in risk of severe hypoxemia between preoxygenation with noninvasive ventilation and high-flow nasal oxygen in critically ill patients with acute hypoxemic respiratory failure. However, preoxygenation with noninvasive ventilation seemed to decrease this risk in moderate-to-severe hypoxemic patients [24]. From this large-scale clinical trial, we performed a post hoc analysis to determine whether critically ill patients with obesity had an increased risk of severe hypoxemia during intubation procedure and whether noninvasive ventilation as compared to high-flow nasal oxygen may prevent severe hypoxemia in this subgroup. Noninvasive ventilation may increase lung volumes thanks to positive pressure and improve oxygenation even more effectively than high-flow nasal oxygen [31], although the latter can provide apneic oxygenation. Therefore, we tested in a post hoc analysis the hypothesis that pre-oxygenation with noninvasive ventilation before intubation as compared with high-flow nasal oxygen may decrease the risk of severe hypoxemia in patients with obesity.

## Method

### Design of the study

This study is a post hoc subgroup analysis of a randomized controlled trial conducted in 28 French ICUs, focusing on the subset of patients with obesity defined by a body mass index at least 30 kg·m^−2^ [24]. The original trial was approved by the independent ethics committee of Poitiers (CPP Ouest III, number 2015-A00530) and registered at http://www.clinicaltrials.gov (NCT02668458). Written informed consent was obtained from all the patients, their next of kin, or another surrogate decision-maker as appropriate. According to French law, this secondary analysis of the original study did not need ethics approval.

### Study population and preoxygenation strategies

In the original trial, patients requiring intubation for acute hypoxemic respiratory failure were randomly assigned to receive preoxygenation by noninvasive ventilation or high-flow nasal oxygen for 3 to 5 min before intubation. All patients had a respiratory rate above 25 breaths per min and a PaO_2_/FiO_2_ ratio below or equal to 300 mm Hg at time of inclusion [24]. This post hoc study focused on the subgroup of patient with obesity defined by a body mass index at least 30 kg m^−2^.

In the noninvasive ventilation group, preoxygenation was carried out via a full-face mask connected to an ICU ventilator, set to pressure-support (PS) mode with a positive end-expiratory pressure (PEEP) of 5 cm H_2_O and FiO_2_ of 100%. Pressure support was adjusted to obtain an expired tidal volume between 6 to 8 mL/kg of predicted bodyweight. Noninvasive ventilation provided oxygenation and ventilation during preoxygenation and from induction up to laryngoscopy, but neither oxygenation nor ventilation during laryngoscopy.

In the high-flow nasal oxygen group, preoxygenation was delivered by continuous oxygen via binasal prongs, with an oxygen flow of 60 L/min through a heated humidifier (MR 850; Fisher & Paykel, Auckland, New Zealand) and FiO_2_ of 100%. Particular attention was paid to perform a jaw thrust so as to maintain patent upper airway and apneic oxygenation during laryngoscopy until the endotracheal tube was placed into the trachea. High-flow nasal oxygen therefore provided oxygenation and little ventilation during preoxygenation and until tracheal intubation was completed.

A protocol of care for the intubation procedure was proposed [25], including at the beginning of the procedure the presence of two operators, systematic fluid loading in the absence of cardiogenic pulmonary edema, then preoxygenation was conducted in a semi-recumbent position at 30° for 3–5 min with the technique assigned by randomization, followed by a rapid-sequence induction using etomidate (0.2–0.3 mg/kg) or ketamine (1.5–3.0 mg/kg), combined with rocuronium (0.6–1.0 mg/kg) or succinylcholine (1.0 mg/kg). In cases of unsuccessful intubation, the following algorithm was proposed (with adaptations for local procedures): an introducer first (intubating stylet or Eschmann introducer), then videolaryngoscopy, an intubation laryngeal mask airway, and finally fiberscopy and rescue percutaneous or surgical tracheostomy. After endotracheal intubation, patients were ventilated with the following settings: a tidal volume of 6 mL/kg of predicted bodyweight, a respiratory rate of 25–30 breaths per min, a positive end-expiratory pressure of 5 cm H_2_O, and a FiO_2_ set to maintain a pulse oximetry above 90%.

### Study outcomes

The primary outcome was the occurrence of an episode of severe hypoxemia, defined by a decreased pulse oximetry below 80% for at least 5 s, during the interval between induction and 5 min after tracheal intubation. To ensure homogeneity of measurement quality of pulse oximetry among participating centers, dedicated portable pulse oximetry monitors (Covidien, Nelcor DS 100A) and single-patient-use digital sensors (Covidien, Max-A-I) were provided to all the participating centers. All values of pulse oximetry were recorded with a 1-Hz frequency from the beginning of preoxygenation to one hour after intubation and reviewed for subsequent analysis by a committee unaware of the study group.

Secondary outcomes included the value of pulse oximetry at the end of preoxygenation and the lowest value during intubation procedure. Other prespecified outcomes included Cormack grade [35], MACOCHA score [36], difficulty for intubation (> 2 laryngoscopic attempts to place the endotracheal tube into the trachea or as lasting more than 10 min using conventional laryngoscopy)[37] and intubation difficulty scale [38].

### Statistical analysis

The analyses were performed as follows: first, in the overall population of the original study, i.e., patients with and without obesity, we performed univariable analyses to compare their characteristics and then multivariable logistic regression analyses to determine independent variables (including obesity) associated with severe hypoxemia; and second in the subgroup of patients with obesity, we performed univariable analyses to compare their characteristics and outcomes according to the strategy of preoxygenation, and multivariable logistic regression analyses to determine factors associated with severe hypoxemia in this subgroup of patients with obesity.

Continuous variables were expressed as mean ± standard deviation or median [interquartile range IQR, 25th to 75th percentiles] when appropriate. Qualitative variables were expressed as frequency and percentage.

Comparisons between patients with and without obesity and comparisons between preoxygenation with noninvasive ventilation or high-flow nasal oxygen in patients with obesity were performed by means of the *χ*^2^ tests or Fisher exact test for categorical variables and Student’s *t*-test or Mann–Whitney test for continuous variables as appropriate. Variables independently associated with severe hypoxemia in the overall population and in the subgroup of patients with obesity were determined by means of multivariable logistic-regression analyses and results are given as odds ratio (OR) with 95% confidence interval (CI). A backward manual selection procedure was performed for the maximal model using all factors associated with outcomes with a *P* value < 0.10. All interactions were tested. The final model included variables significantly associated with severe hypoxemia. A two-tailed p value of less than 0.05 was considered as statistically significant. All analyses were performed using SAS software, version 9.2 (SAS Institute Cary, NC).

## Results

### Comparison between patients with and without obesity

**Fig. 1:**
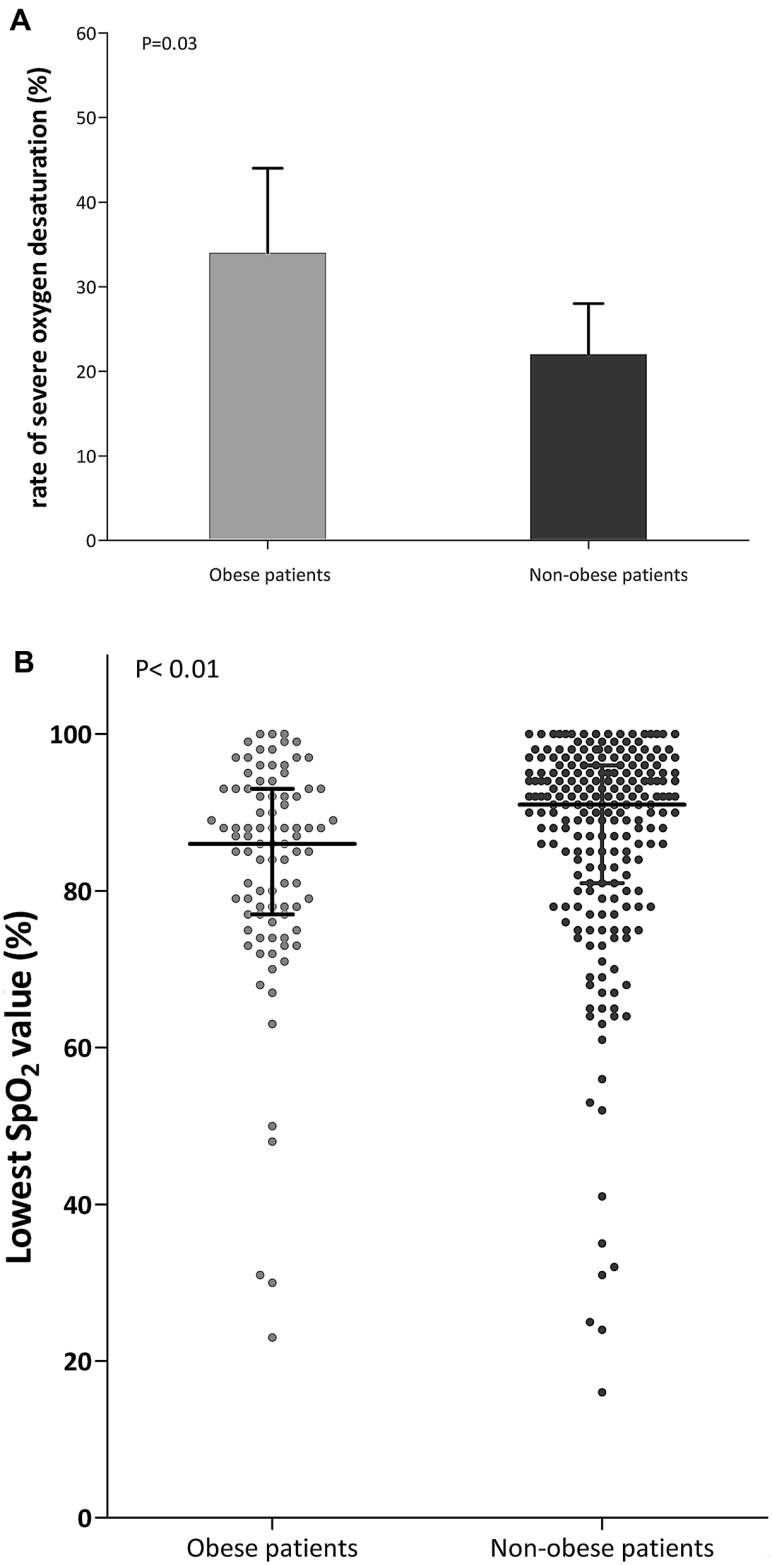
**A** Rates of severe hypoxemia during intubation procedure after preoxygenation using noninvasive ventilation or high-flow nasal cannula oxygen therapy in patients with obesity (grey bar) and without obesity (dark bar). **B** Lowest individual pulse oximetry values during intubation procedure after preoxygenation using noninvasive ventilation or high-flow nasal cannula oxygen therapy in patients with obesity (grey points) and without obesity (dark points)

Among the 313 patients included in the original trial, 91 (29%) patients had obesity with a mean body mass index of 35 ± 5 kg·m^−2^. Patients with obesity had an increased risk of difficult intubation, as assessed by higher proportion of patients with MACOCHA score ≥ 3 or Cormack grade III or IV (Table 1). Patients with obesity were more likely to experience an episode of severe hypoxemia during intubation procedure than patients without obesity: 34% (31 out of 91 patients) vs. 22% (49 out of 222) (difference, 12%; 95% CI 1 to 23%; *P* = 0.03) (Fig. 1). The minimal pulse oximetry value during intubation procedure was significantly lower in patients with obesity than in patients without obesity: 86% in median [IQR, 77 to 93] versus 91% [IQR, 81 to 96], *P* < 0.01 (Fig. 1). Similarly, pulse oximetry at the end of pre-oxygenation was lower in patients with obesity than in patients without obesity (*P* = 0.04). There was no difference in immediate and late complications between patients with or without obesity. After multivariable logistic-regression analysis, the three factors independently associated with severe hypoxemia during intubation procedure in overall population were obesity (OR 2.14; 95% CI 1.18 to 3.87; *P* = 0.012), intubation difficulty scale > 5 points (OR 4.0; 95% CI 1.90 to 8.76, *P* = 0.0003) and PaO_2_/FiO_2_ ratio at baseline (OR 0.99; 95% CI 0.98 to 0.99, *P* < 0.0001) (Additional file 1: Tables S1, S2).

**Table 1:** Baseline characteristics of the intention-to-treat population according to obese status

	Obese patients with obesity (*n* = 91)	Non-obese patients without obesity (*n* = 222)	*P* value
Characteristics of the patients			
Age, year, mean ± SD	66 ± 14	63 ± 14	0.13
Male sex, *n* (%)	64 (70)	148 (67)	0.53
Body mass index,^a^ kg·m^−2^, mean ± SD	35 ± 5	24 ± 3	< 0.0001
SAPS II^b^, point, mean ± SD	49 ± 19	52 ± 19	0.20
Reason for ICU admission			0.14
Respiratory primary failure, *n* (%)			
Respiratory infection	28 (31)	82 (37)	
COPD exacerbation	8 (9)	8 (4)	
Extra-pulmonary ARDS	3 (3)	3 (1)	
Pulmonary atelectasis	2 (2)	2 (1)	
Other	6 (7)	27 (12)	
Non-respiratory primary failure, *n* (%)			
Shock	19 (21)	47 (21)	
Cardiogenic pulmonary edema	4 (4)	13 (6)	
Neurologic	7 (8)	6 (3)	
Other	12 (13)	24 (11)	
Post-operative, *n* (%)	2 (2)	10 (4)	
Clinical characteristics			
Oxygen device the last hour before inclusion, *n* (%)			0.48
Standard oxygen	38 (42)	98 (44)	
High-flow nasal cannula oxygen	28 (31)	77 (35)	
Non-invasive ventilation	25 (27)	47 (21)	
Vasopressor support at inclusion, *n* (%)	19 (21)	43 (19)	0.76
Bilateral pulmonary infiltrates, *n* (%)	54 (71)	140 (73)	0.71
Respiratory variables			
Respiratory rate, breaths·min, mean ± SD	30 ± 7	31 ± 8	0.50
PaO_2_/FIO_2_ ratio, mm Hg, mean ± SD	152 ± 65	142 ± 68	0.26
MACOCHA score_,_^c^ *n* (%)			0.003
< 3	68 (75)	195 (88)	
≥ 3	23 (25)	26 (12)	
Cormack III or IV,^d^ *n* (%)	15 (16)	14 (6)	0.005
Outcomes			
SpO_2_ < 80% during intubation procedure, *n* (%)	31 (34)	49 (22)	0.03
Lowest SpO_2_ during intubation procedure, %, median (IQR)	86 (77- 93)	91 (81–96)	0.002
SpO_2_ at the beginning of preoxygenation, %, median (IQR)	96 (92–98)	95 (92–99)	0.82
SpO_2_ at the end of preoxygenation, %, median (IQR)	99 (97–100)	100 (98–100)	0.04
Intubation Difficulty Scale,^e^ *n* (%)			0.29
≤ 5	75 (85)	196 (89)	
> 5	13 (15)	23 (11)	
Immediate complications, *n* (%)			
Hypotension	41 (45)	115 (52)	0.28
Sustained cardiac arrhythmia	0	6 (3)	0.19
Bradycardia or cardiac arrest during and after intubation	5 (5)	6 (3)	0.22
Esophageal intubation	6 (7)	8 (4)	0.24
Regurgitation	0	2 (1)	0.99
Gastric distension	3 (3)	14 (6)	0.26
Dental injury	0 (0)	1 (0)	0.99
Agitation	0 (0)	1 (0)	0.99
New infiltrate on chest-ray after intubation	14 (18)	47 (25)	0.20
Late outcomes			
Ventilator-associated pneumonia during ICU stay, *n* (%)	22 (24)	44 (20)	0.39
Death at day 28	36 (40)	75 (34)	0.33
SOFA score at Day 1, points, mean ± SD	9 ± 4	8 ± 4	0.19
SOFA score at Day 7, points, mean ± SD	6 ± 4	5 ± 3	0.48
Duration of mechanical ventilation, days, median (IQR)	9 (5–17)	7 (4–16)	0.46
Ventilator-free day at day 28, median (IQR)	5 (0–19)	8 (0–22)	0.26
ICU length of stay, days, median (IQR)	11 (6–20)	10 (6–17)	0.27

*COPD* chronic obstructive pulmonary disease, *ARDS* acute respiratory distress syndrome, *SpO*_*2*_ pulse oximetry, *SD* standard deviation, *SOFA* Sepsis-related Organ Failure Assessment, *ICU* intensive care unit

^a^The body mass index is the weight in kilograms divided by the square of the height in meters

^b^The Simplified Acute Physiology Score (SAPS) II is calculated from 17 variables at inclusion, information about previous health status, and from information obtained at admission. Scores can range from 0 to 163, with higher scores indicating more severe disease

^c^MACOCHA is calculated from 7 variables including Mallampati score III or IV, apnea syndrome, cervical spine limitation, opening mouth less than 3 cm, coma, hypoxia, non-trained operator. Score range from 0 to 12 points, with higher scores indicating risk of difficult intubation

^d^Cormack grade III, if no part of the glottis can be seen, but only the epiglottis, grade IV, if not even the epiglottis can be exposed

^e^The Intubation Difficulty Scale denotes the Intubation Difficulty Scale score, 0 easy, 0 to 5 slight difficulty, > 5 moderate to major difficulty for intubation

### Comparison between preoxygenation with noninvasive ventilation or high-flow nasal oxygen in patients with obesity

Among the 91 patients with obesity, 40 patients received noninvasive ventilation during preoxygenation and 51 patients received high-flow nasal oxygen. Characteristics of the patients at baseline did not differ between the two groups (Table 2). Preoxygenation lasted 4 min in median [IQR, 4 to 5] with noninvasive ventilation and 4 min [IQR, 4 to 5] with high-flow nasal oxygen (*P* = 0.82). The ventilator settings with noninvasive ventilation were a PS level of 9 ± 4 cm H_2_O, PEEP of 5 ± 0.5 cm of H_2_O, and FiO_2_ of 0.99 ± 0.06, resulting in an expired tidal volume of 7.9 ml ± 2.5 ml/kg of predicted body weight. High-flow nasal oxygen was delivered with a gas flow of 58 ± 9 L/min with FiO_2_ of 0.99 ± 0.08. Preoxygenation was discontinued in one patient during preoxygenation with high-flow nasal oxygen due to severe hypoxemia during the procedure, while preoxygenation with noninvasive ventilation was not discontinued.

**Table 2:** Baseline characteristics of obese patients with obesity according to the strategy of preoxygenation

	Non-invasive ventilation (*n* = 40)	High-flow nasal cannula oxygen (*n* = 51)	*P* value
Demographic characteristics			
Age, year, mean ± SD	66 ± 12	66 ± 16	0.85
Male sex, *n* (%)	29 (73)	35 (69)	0.69
Body mass index,^a^ kg·m^−2^, mean ± SD	35 ± 5	34 ± 4	0.28
SAPS II^b^, point, mean ± SD	50 ± 21	49 ± 17	0.86
Reason for ICU admission			0.98
Respiratory primary failure, *n* (%)			
Respiratory infection	12	16	
COPD exacerbation	4	4	
Extra-pulmonary ARDS	2	1	
Pulmonary atelectasis	1	1	
Other	3	3	
Non-respiratory primary failure, *n* (%)			
Shock	8	11	
Cardiogenic pulmonary edema	1	3	
Neurologic	4	3	
Other	4	8	
Post-operative, *n* (%)	1	1	
Clinical characteristics			
Oxygen device the last hour before inclusion, *n* (%)			0.17
Standard oxygen	19 (47)	19 (37)	
High-flow nasal cannula oxygen	14 (35)	14 (27)	
Non-invasive ventilation	7 (18)	18 (35)	
Vasopressor support at inclusion, *n* (%)	7 (18)	12 (24)	0.48
Bilateral pulmonary infiltrates, *n* (%)	22 (65)	32 (76)	0.27
Respiratory variables			
Respiratory rate, breaths/min	30 ± 7	30 ± 8	0.71
PaO_2_/FIO_2_ ratio, mm Hg	149 ± 65	154 ± 66	0.72
MACOCHA score, ^c^ *n* (%)			0.36
< 3	28 (70)	40 (78)	
≥ 3	12 (30)	11 (22)	
Cormack III or IV, ^d^ *n* (%)	(1, 2)9 (23)	6 (12)	0.17

*COPD* chronic obstructive pulmonary disease, *SD* standard deviation

^a^The body mass index is the weight in kilograms divided by the square of the height in meters

^b^The Simplified Acute Physiology Score (SAPS) II is calculated from 17 variables at inclusion, information about previous health status, and from information obtained at admission. Scores can range from 0 to 163, with higher scores indicating more severe disease

^c^MACOCHA is calculated from 7 variables including Mallampati score III or IV, apnoea syndrome, cervical spine limitation, opening mouth less than 3 cm, coma, hypoxia, non-trained operator. Score range from 0 to 12 points, with higher scores indicating risk of difficult intubation

^d^Cormack grade III, if no part of the glottis can be seen, but only the epiglottis, grade IV, if not even the epiglottis can be exposed

### Primary outcome: episodes of severe hypoxemia

**Fig. 2:**
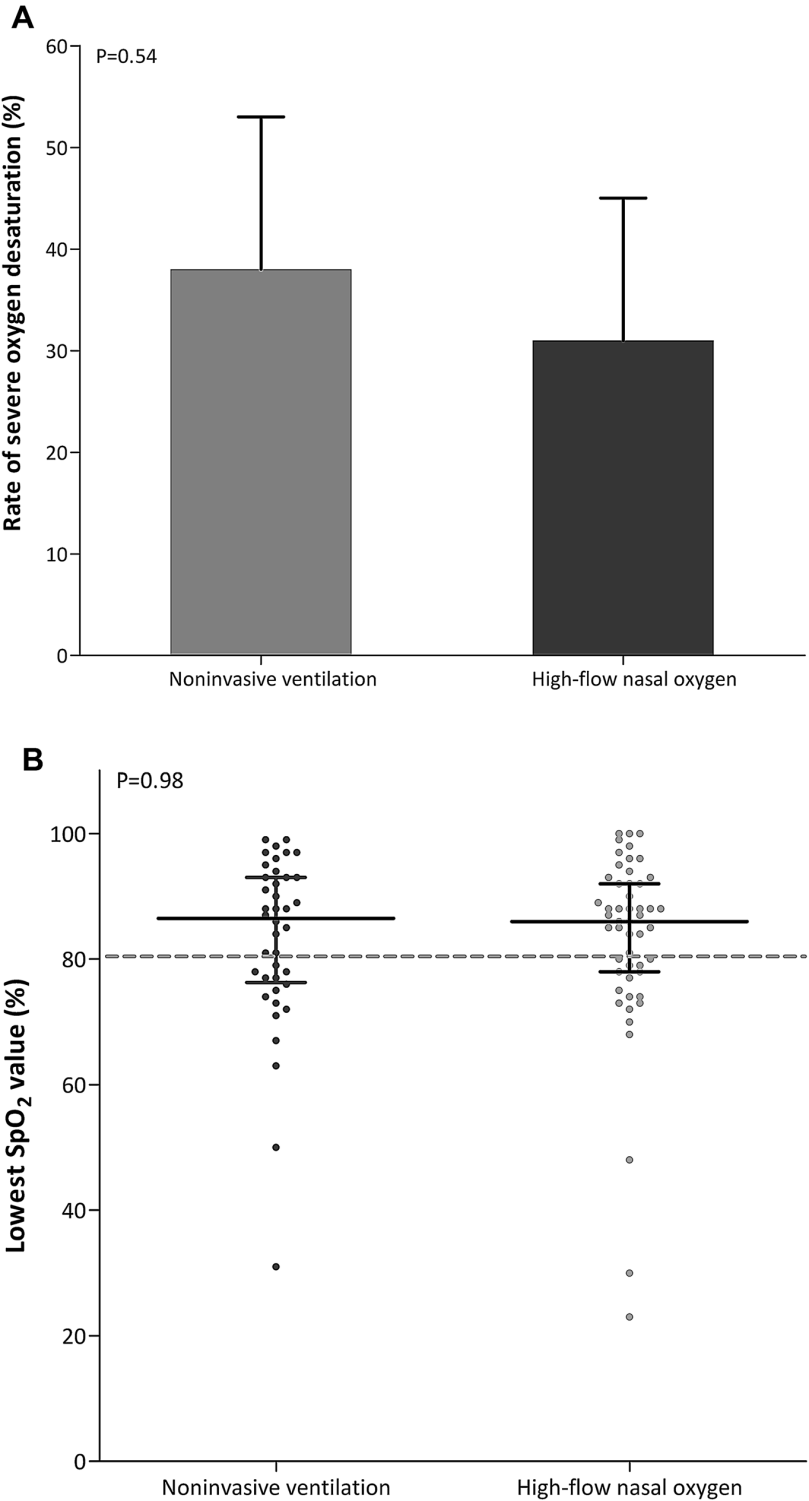
**A** Rates of severe hypoxemia during intubation procedure in patients with obesity after preoxygenation with non-invasive ventilation (grey bar) and high-flow nasal cannula oxygen therapy (dark bar). **B** Lowest individual pulse oximetry values during intubation procedure after preoxygenation with noninvasive ventilation (grey points) and high-flow nasal cannula oxygen therapy (dark points) in patients with obesity

Severe hypoxemia occurred in 15 of 40 patients (37%) after pre-oxygenation with noninvasive ventilation and 16 of 51 patients (31%) with high-flow nasal oxygen (difference, 6%; 95% CI − 13 to 25%; *P* = 0.54) (Table 3, Fig. 2).

**Table 3:** Primary and secondary outcomes in obese patients with obesity according to the strategy of preoxygenation

	Non-invasive ventilation (*n* = 40)	High-flow nasal cannula oxygen (*n* = 51)	*P* value
Outcomes			
SpO_2_ < 80% during intubation procedure, n (%)	15 (37)	16 (31)	0.54
Lowest SpO_2_ during intubation procedure, median (IQR)	87 (77–93)	86 (78–92)	0.98
SpO_2_ at the beginning of preoxygenation, %, median (IQR)	94 (92–99)	96 (93–99)	0.25
SpO_2_ at the end of preoxygenation, %, median (IQR)	99 (98–100)	99 (96–100)	0.26
Procedure of tracheal intubation, *n* (%)			
Duration of laryngoscopy, *n* (%)			0.98
< 1 min	24 (62)	31 (61)	
1 to 3 min	10 (26)	14 (27)	
> 3 min	5 (13)	6 (12)	
Number of laryngoscopy attempt			0.90
One	30 (75)	38 (79)	
Two	8 (20)	8 (17)	
Three or more, or > 10 min	2 (5)	2 (4)	
First junior operator	10 (25)	12 (24)	0.87
Intervention of another skilled operator	14 (35)	12 (23)	0.23
Use of alternative management devices	7	8	0.81
Introducer	6 (15)	8 (16)	
Other	1 (3)	0	
Intubation Difficulty Scale,^a^ *n* (%)			0.35
≤ 5	30 (81)	45 (88)	
> 5	7 (19)	6 (12)	

^a^The Intubation Difficulty Scale denotes the Intubation Difficulty Scale score, 0 easy, 0 to 5 slight difficulty, > 5 moderate to major difficulty for intubation

*SpO*_*2*_ pulse oximetry, *IQR* interquartile range

### Secondary outcomes

Lowest pulse oximetry values during intubation procedure were 87% [IQR, 77 to 93] after preoxygenation with noninvasive ventilation and 86% [IQR, 78 to 92] with high-flow nasal oxygen (*P* = 0.98). Pulse oximetry at the end of preoxygenation, duration of laryngoscopy or procedure of intubation did not differ between the two groups (Table 3, Fig. 2).

### Factors associated with severe hypoxemia in patients with obesity

After univariable analysis, the three factors significantly associated with severe hypoxemia in patients with obesity were intubation difficulty scale > 5 points, respiratory primary failure as reason for admission, and PaO_2_/FiO_2_ ratio at randomization (Additional file 1: Table S3). After multivariable logistic regression analysis with a maximal model forced with strategy of preoxygenation, the two factors independently associated with severe hypoxemia were intubation difficulty scale > 5 points (OR 7.9; 95% CI 1.9 to 33.6; *P* = 0.005) and respiratory primary failure as reason for admission (OR 5.8; 95% CI 1.9 to 17.5; *P* = 0.002) (Additional file 1: Table S4).

## Discussion

This post hoc analysis showed that obesity was an independent factor associated with severe hypoxemia during intubation procedure of patients with acute respiratory failure (defined as a PaO_2_/FiO_2_ ratio equal or below 300 mm Hg) requiring intubation in ICU. In the subgroup of patients with obesity (defined as a BMI at least 30 kg/m^2^), preoxygenation using noninvasive ventilation did not seem to decrease this risk of severe hypoxemia compared with preoxygenation using high-flow nasal oxygen. However, an intubation difficulty scale score above 5 points, i.e., difficulties during intubation, and respiratory failure as reason for ICU admission were the two factors strongly associated with increased risk of severe hypoxemia during intubation procedure in patients with obesity.

### Risk of severe hypoxemia in patients with obesity

Observational studies conducted in operating room reported that patients with obesity as compared patients without obesity had a reduced time to desaturation (pulse oximetry < 90%) inversely proportional to body mass index [11]. Critically ill patients with obesity have a higher rate of severe hypoxemia (pulse oximetry < 80%) during intubation procedure reaching 17% vs. 0% in patients with obesity intubated in operating room as reported in a large-scale observational study [4]. Hypoxemia in obesity may be explained by a reduced ventilation–perfusion ratio due to atelectasis, especially during general anesthesia [39, 40], which leads to subsequent reduction in functional residual capacity [9]. This is initiated by two mechanisms the supine position and compression atelectasis caused by an increased intra-abdominal pressure and leading to compression of the thoracic cavity and airway closure [41]. As a result, the increased physiologic dead space causes shunting of blood through non-ventilated lung tissue, subsequently increased venous admixture and finally hypoxemia [42]. Aside from these physiological abnormalities, the phenomenon of atelectasis formation can be aggravated by the use of high fraction of inspired oxygen, resulting in absorption atelectasis, as is the case during the intubation procedure, and can thereby enhance the risk of severe hypoxemia [43]. Moreover, difficult intubation occurring in around 15% of critically ill patients with obesity [3, 5] may also contribute to severe hypoxemia [4]. In our study, we observed similar rates of difficult intubation in the subgroup of patients with obesity, as assessed by an intubation difficulty scale score above 5 points [38]. However, the rate of severe hypoxemia was higher, a finding explained by the inclusion of patients with obesity with acute hypoxemic respiratory failure and accurate offline analysis of pulse oximetry recordings throughout the intubation procedure, which may have identified otherwise unrecognized events.

### Efficacy of preoxygenation strategies

In operating rooms, noninvasive ventilation and high-flow nasal oxygen have been evaluated in patients with obesity as alternative strategies to standard preoxygenation using valve-bag mask, with the aim of optimizing intubation procedure [44–48]. In 27 patients with morbid obesity, noninvasive ventilation set with a PEEP of 10 cm H_2_O resulted in an approximately 50% increase of oxygenation level and apnea time [46]. Another physiological study conducted in 66 patients with morbid obesity showed that in addition to increase oxygenation, noninvasive ventilation with a 6–8 cm H_2_O PEEP level, improved lung volumes or alveolar recruitment [44]. Likewise, PEEP application prevented atelectasis formation as compared to preoxygenation with PS without PEEP [39]. Indeed, PEEP contributes to increase functional residual capacity, which is the main oxygen store of the body, and this factor may explain its superiority to standard preoxygenation using valve-bag mask.

High-flow nasal oxygen also helps to improve intubation procedure in operating rooms. A randomized controlled study including 40 patients with obesity showed that preoxygenation with high-flow nasal oxygen as compared to valve-bag mask resulted in higher minimal value of pulse oximetry during the procedure and increased apnea time of 40% before desaturation [48]. Therefore, high-flow nasal oxygen has rapid effects similar to noninvasive ventilation thanks to PEEP effect [30, 32], in a lower magnitude [31], and also the ability to provide high inspired fraction of oxygen [29, 49]. However, the main expected physiological effect during preoxygenation with high-flow nasal oxygen is apneic oxygenation, which is particularly relevant in patients with obesity [34, 50, 51]. High-flow nasal oxygen can provide oxygenation during the preoxygenation phase and from induction to tracheal intubation, thereby leading to higher efficacy during an intubation procedure as compared to standard preoxygenation.

To date, only one randomized, controlled trial including 100 patients with obesity has compared preoxygenation with high-flow nasal oxygen and noninvasive ventilation before intubation in operating room. The results were in favor of noninvasive ventilation with better oxygenation and fewer episodes of hypoxemia [52]. In critically ill patients with obesity, data on effects of preoxygenation strategies are very scarce. However, noninvasive ventilation [24, 27, 28] or positive pressure [53] applied during preoxygenation in unselected patients with acute hypoxemic respiratory failure seems to be more beneficial as compared to standard oxygen or high-flow nasal oxygen in terms of minimal pulse oximetry or occurrence of severe hypoxemia during intubation procedure.

Although our original trial showed benefits of noninvasive ventilation as compared to high-flow nasal oxygen during preoxygenation in moderate-to-severe hypoxemic patients [24], the present post hoc analysis did not find any difference between noninvasive ventilation and high-flow nasal oxygen in the subset of patients with obesity and acute hypoxemic respiratory failure*.* There are several potential explanations for these discrepancies between results in operating room and those in critically ill patients. First, previous studies conducted in operating rooms included patients with obesity without acute respiratory failure or hypoxemia. In patients with primary respiratory failure as reason for intubation, provision of oxygen by noninvasive ventilation or high-flow nasal oxygen was insufficient to avert intubation and improve hypoxemia, which meant that continuing noninvasive ventilation or high-flow nasal oxygen might be expected to be similarly ineffective.

Second, patients may have benefited from apneic oxygenation with high-flow nasal oxygen but not with noninvasive ventilation, and patients receiving noninvasive ventilation might have experienced longer apnea between onset of muscle relaxation and intubation than patients receiving high-flow nasal oxygen. This prolonged apnea could have hastened desaturation during intubation after noninvasive ventilation. During this same time, apneic oxygenation in patients receiving high-flow oxygen could have delayed desaturation and offset the benefit of noninvasive ventilation. At last, the PEEP level applied in our study, around 5 cm of H_2_O, could be inadequate to enable alveolar recruitment in a population of patients with obesity and an underlying lung disease [44, 46]. Indeed, applying higher PEEP levels, up to 12 cm H_2_O, has been shown to be feasible and well tolerated in critically ill patients with acute hypoxemic respiratory failure [54, 55]. However, this has been carried out with helmet NIV, which may avoid leakage and improve tolerance [56]. An alternative to reduce leakage with noninvasive ventilation through face mask by applying high PEEP levels, could be to set pressure support at the minimum level. Although a low level of PEEP has been shown to prevent atelectasis formation in patients scheduled for elective surgery [39], a physiological study showed that a higher PEEP level of at least of 10 cm of H_2_O through NIV with face mask was necessary and more efficient than 5 cm H_2_O as a mean of obtaining alveolar recruitment in obese patients [57]. Obviously, this way of applying noninvasive ventilation with a higher level of PEEP during preoxygenation needs to be confirmed by clinical studies, especially in critically ill patients with obesity and acute hypoxemic respiratory failure.

### Clinical implications and limitations

The main limitations of our study were the post hoc nature of the analysis and the small numbers of patients in each subgroup, which could lead to a lack of power to detect subgroup effects. However, the characteristics of patients were similar in both groups and rates of difficulty during intubation were similar to those previously reported [4, 10]. Second, a strategy of preoxygenation with valve-bag mask was not considered in the original study, given the results of previous studies showing superiority of high-flow nasal oxygen [23, 26], which has been also reported in operating room for patients with obesity [47, 48].

## Conclusion

In summary, patients with obesity and acute hypoxemic respiratory failure have a higher risk of severe hypoxemia during intubation procedure as compared to patients without obesity, but preoxygenation with noninvasive ventilation may not reduce this risk compared with high-flow nasal oxygen therapy.

## Supplementary Information

**Additional file 1: Table S1.** Comparison of characteristics in patients with and without severe hypoxemia during intubation procedure. **Table S2.** Multivariable logistic regression analyses of factors associated with severe hypoxemia during intubation procedure. **Table S3.** Comparison of Characteristics in Patients with Obesity and Severe Hypoxemia during Intubation Procedure. **Table S4.** Multivariable logistic regression analyses of factors associated with severe hypoxemia during intubation procedure in patients with obesity.

## Data Availability

The datasets analyzed during the current study are available from the corresponding author on reasonable request and after submission to the scientist committee of the study.

## Abbreviations

ICU: Intensive care unit
PEEP: Positive end-expiratory pressure
FiO_2_: Fraction of inspired oxygen
PaO_2_: Partial pressure of arterial oxygen
PS: Pressure support
IQR: Interquartile range
