# Supplementary material for: Noninvasive ventilation vs. high-flow nasal cannula oxygen for preoxygenation before intubation in patients with obesity: a post hoc analysis of a randomized controlled trial
Source: Ann Intensive Care. 2021 Jul 22;11:114. doi: 10.1186/s13613-021-00892-8 (PMC8295638; doi:10.1186/s13613-021-00892-8)
Supplement: Supplementary file 1 — Additional file 1: Table S1. Comparison of characteristics in patients with and without severe hypoxemia during intubation procedure. Table S2. Multivariable logistic regression analyses of factors associated with severe hypoxemia during intubation procedure. Table S3. Comparison of Characteristics in Patients with Obesity and Severe Hypoxemia during Intubation Procedure. Table S4. Multivariable logistic regression analyses of factors associated with severe hypoxemia during intubation procedure in patients with obesity. [file 13613_2021_892_MOESM1_ESM.docx]

**ADDITIONAL MATERIAL**

**Effect of Noninvasive Ventilation and High-Flow Nasal Cannula Oxygen Therapy during preoxygenation in patients with obesity: a post-hoc analysis of a randomized controlled trial.**

*Maeva Rodriguez, MD, Stéphanie Ragot, PharmD, PhD, Rémi Coudroy, PhD, Jean-Pierre Quenot, MD, PhD, Philippe Vignon, MD, PhD, Jean-Marie Forel, MD, Alexandre Demoule, MD, PhD, Jean-Paul Mira, MD, PhD, Jean-Damien Ricard, MD, PhD, Saad Nseir, PhD, Gwenhael Colin, MD, Bertrand Pons, MD, Pierre Eric Danin, MD, Jérome Devaquet, MD, Gwenael Prat, MD, Hamid Merdji, MD, Franck Petitpas, MD, Emmanuel Vivier, MD, PhD, Armand Mekontso-Dessap, MD, PhD, Mai-Anh Nay, MD, Pierre Asfar, MD, PhD, Jean Dellamonica, MD, PhD, Laurent Argaud, MD, PhD, Stephan Ehrmann, MD, PhD, Muriel Fartoukh, MD, PhD, Christophe Girault, MD, René Robert, MD, PhD, Arnaud W. Thille, MD, PhD, Jean-Pierre Frat, MD, PhD, and REVA network.*

| Table S1. Comparison of characteristics in patients with and without severe hypoxemia during intubation procedure. | | | |
| --- | --- | --- | --- |
| Variables | **SpO_2_<80%**  **(n = 80)** | **SpO_2_>= 80%**  **(n = 233)** | ***P* value** |
| Age, year | 63 ± 15 | 64 ± 13 | 0.34 |
| Male sex, no. (%) | 49 (61) | 163 (70) | 0.15 |
| Body-mass index, kg·m^-2^ | 28 ± 7 | 26 ± 6 | 0.009 |
| Obesity, no. (%) | 31 (39) | 60 (26) | **0.027** |
| SAPS II | 49 ± 18 | 52 ± 19 | 0.23 |
| SOFA at inclusion, points | 5 ± 3 | 5 ± 3 | 0.31 |
| Chronic respiratory failure, no. (%) | 23 (29) | 63 (27) | 0.77 |
| Respiratory rate at randomization, breaths·min^-1^ | 31 ± 8 | 30 ± 9 | 0.68 |
| Arterial blood gas |  |  |  |
| PaO_2_ at randomization, mm Hg | 78 ± 28 | 89 ± 35 | 0.013 |
| FIO_2_ at randomization | 0.75 ± 0.23 | 0.64 ± 0.24 | 0.0003 |
| PaO_2_/FIO_2_ ratio at randomization – mm Hg | 117 ± 59 | 155 ± 68 | **<0.0001** |
| MACOCHA score, no of patients/total no. |  |  | 0.045 |
| <3 | 61 (77) | 202 (87) |  |
| ≥3 | 18 (23) | 31 (13) |  |
| IDS score, no of patients/total no. |  |  | **<0.0001** |
| ≤5 | 58 | 213 |  |
| >5 | 19 | 17 |  |

SAPS II, Simplified Acute Physiology Score is calculated from 17 variables at inclusion, information about previous health status, and from information obtained at admission. Scores can range from 0 to 163, with higher scores indicating more severe disease.

SOFA, Sepsis-related Organ Failure Assessment (SOFA) score range from 0 to 24, with higher scores indicating more severe organ failure.

MACOCHA is calculated from 7 variables including Mallampati score III or IV, apnea syndrome, cervical spine limitation, opening mouth less than 3 cm, coma, hypoxia, non-trained operator. Score range from 0 to 12 points, with higher scores indicating risk of difficult intubation.

IDS, Intubation Difficulty Scale denotes the Intubation Difficulty Scale score, 0 easy, 0 to 5 slight difficulty, >5 moderate to major difficulty for intubation.

| Table S2. Multivariable logistic regression analyses of factors associated with severe hypoxemia during intubation procedure. | | | |
| --- | --- | --- | --- |
|  | **Odd ratio** | **95% CI** | ***P* value** |
| Obesity | 2.14 | 1.18-3.87 | 0.012 |
| PaO_2_/FIO_2_ ratio at randomization | 0.99 | 0.98-0.99 | < 0.0001 |
| IDS score >5 | 4.08 | 1.90-8.76 | 0.0003 |

IDS, Intubation Difficulty Scale denotes the Intubation Difficulty Scale score, 0 easy, 0 to 5 slight difficulty, >5 moderate to major difficulty for intubation.

| Table S3. Comparison of Characteristics in Patients with Obesity and Severe Hypoxemia during Intubation Procedure. | | | |
| --- | --- | --- | --- |
| Variables | **SpO_2_<80%**  **(n=31)** | **SpO_2_>=80%**  **(n=60)** | ***P* value** |
| Age, year | 63 ± 15 | 67 ± 13 | 0.12 |
| Male sex, n (%) | 20 (64) | 44 (73) | 0.38 |
| Body-mass index, kg·m^-2^ | 35 ± 6 | 34 ± 4 | 0.22 |
| SAPS II, point | 46 ± 15 | 51 ± 20 | 0.20 |
| SOFA at inclusion, point | 5 ± 3 | 7 ± 4 | 0.03 |
| Chronic respiratory failure, n (%) | 9 (29) | 20 (33) | 0.68 |
| Sleep apnea | 5 (16) | 9 (15) | 0.89 |
| Reason for ICU admission, n (%) |  |  | **0.002** |
| Respiratory primary failure | 23 (74) | 24 (40) |  |
| Respiratory infection | 16(52) | 12(20) |  |
| COPD exacerbation | 2(6) | 6(10) |  |
| Extra-pulmonary ARDS | 0(0) | 3(5) |  |
| Pulmonary atelectasis | 1(3) | 1(2) |  |
| Other | 4(13) | 2(3) |  |
| Non-respiratory primary failure | 8 (26) | 36 (60) |  |
| Shock | 4(13) | 15(25) |  |
| Cardiogenic pulmonary oedema | 1(3) | 3(5) |  |
| Neurologic | 2(6) | 5(8) |  |
| Other | 1(3) | 11(18) |  |
| Post-operative | 0(0) | 2(3) |  |
| Vasopressor support at inclusion, n (%) | 5 (16) | 14 (23) | 0.42 |
| Respiratory rate, breaths·min^-1^ | 30 ± 8 | 30 ± 7 | 0.82 |
| PaO_2_:FIO_2_ ratio, mm Hg | 128 ± 62 | 164 ± 64 | **0.01** |
| MACOCHA score, n (%) |  |  | 0.27 |
| <3 | 21 (68) | 47 (78) |  |
| ≥3 | 10 (32) | 13 (22) |  |
| Cormack III or IV, n of patient/total, n (%) | 8 (26) | 7 (12) | 0.08 |
| Intubation Difficulty Scale, n (%) |  |  | **0.004** |
| ≤5 | 21 (70) | 54 (93) |  |
| >5 | 9 (30) | 4 (7) |  |

SAPS II, Simplified Acute Physiology Score is calculated from 17 variables at inclusion, information about previous health status, and from information obtained at admission. Scores can range from 0 to 163, with higher scores indicating more severe disease.

SOFA, Sepsis-related Organ Failure Assessment (SOFA) score range from 0 to 24, with higher scores indicating more severe organ failure.

MACOCHA is calculated from 7 variables including Mallampati score III or IV, apnea syndrome, cervical spine limitation, opening mouth less than 3 cm, coma, hypoxia, non-trained operator. Score range from 0 to 12 points, with higher scores indicating risk of difficult intubation.

IDS, Intubation Difficulty Scale denotes the Intubation Difficulty Scale score, 0 easy, 0 to 5 slight difficulty, >5 moderate to major difficulty for intubation.

| Table S4. Multivariable logistic regression analyses of factors associated with severe hypoxemia during intubation procedure in patients with obesity. | | | |
| --- | --- | --- | --- |
|  | **Odd ratio** | **95% CI** | ***P* value** |
| Respiratory primary failure | 5.8 | 1.90-17.47 | 0.002 |
| IDS score >5 | 7.9 | 1.85-33.56 | 0.005 |

IDS, Intubation Difficulty Scale denotes the Intubation Difficulty Scale score, 0 easy, 0 to 5 slight difficulty, >5 moderate to major difficulty for intubation.
